# Notes on the genus *Microcriodes* Breuning, with description of a new species from Xizang, China (Coleoptera, Cerambycidae, Lamiinae, Batocerini)

**DOI:** 10.3897/zookeys.412.7585

**Published:** 2014-05-29

**Authors:** Wen-Xuan Bi, Mei-Ying Lin

**Affiliations:** 1 Key Laboratory of Zoological Systematics and Evolution, Institute of Zoology, Chinese Academy of Sciences, Beichen West Road, Chaoyang, Beijing, 100101, China; 2 Room 401, No. 2, Lane 155, Lianhua South Road, Shanghai, 201100 China

**Keywords:** *Microcriodes*, new record, new species, taxonomy, Oriental region

## Abstract

The genus *Microcriodes* Breuning is newly recorded from China upon the discovery of *M. sikkimensis* Breuning, 1943 and *M. wuchaoi* **sp. n.** from Motuo, Southeast Xizang. Illustrations of the habitus, genitalia including non-everted endophallus, as well as diagnostic features are provided.

## Introduction

The genus *Microcriodes* was established by Breuning (1943) on the basis of an Indian species, *Microcriodes sikkimensis* Breuning, 1943. Gilmour and Dibb (1948) only referred to Breuning’s original description in their revision of the Batocerini, but Gilmour (1963) redescribed the species based on the holotype and an additional pair of specimens. The locality of one male specimen mentioned by Gilmour (1963) was written in question as China (probably erroneous). Since then, few people have referred to this genus expect for Weigel (2012), who reported *Microcriodes sikkimensis* from Arunachal Pradesh, India, and Tavakilian and Chevillotte (2014), who summarized all related information.

From 2010 to 2013, the first author and his team made several expeditions to Southeast mountainous region of Xizang and brought back a large number of cerambycid beetles. In the course of the identification work of these specimens, we found a species which was identical to *Microcriodes sikkimensis* and another similar congener.

In this paper, we describe it here as a second new species, *Microcriodes wuchaoi* sp. n., and simultaneously review *Microcriodes sikkimensis* with supplementary descriptions and notes on the variations. The male genitalia including non-everted endophallus of both species are figured to facilitate the comparison. This is the first formal record of *Microcriodes* from China though a doubtful record was presented by Gilmour (1963).

Materials are deposited in the following institutions, museums or private collections; abbreviations as shown in the text:

CBWX Collection of Wen-Xuan Bi, Shanghai, China

CCCC Collection of Chang-Chin Chen, Tianjin, China

IZAS Institute of Zoology, Chinese Academy of Sciences, Beijing, China

NHML The Natural History Museum, London, UK

SNUC Insect Collection of Shanghai Normal University, Shanghai, China

The following abbreviations for terminology of endophallic structures are used in the text: BPH-basal phallomere; MPH-median phallomere; APH-apical phallomere; MT-medial tube; CT-central trunk; PB-preapical bulb.

## Results

### Microcriodes

Breuning, 1943

http://species-id.net/wiki/Microcriodes

Microcriodes Breuning, 1943: 14. Type species: *Microcriodes sikkimensis* Breuning, 1943, by monotypy.
Microcriodes ; Gilmour and Dibb 1948: 99; Gilmour 1963: 483, pl. 2, figs 4-5; Rigout 1982: 10, pl. 14; Nýlander 2004: 249.

#### Redescription

(Breuning 1943; Gilmour and Dibb 1948; partly modified). Body elongate. Eyes coarsely faceted, strongly emarginate; lower lobe large, distinctly longer than width. Frons wider than long. Antennal tubercles widely separated, moderately raised. Antennae long and smooth, more than 1.8 times (in male) or 1.1–1.5 times (in female) as long as body length; basal 3 antennomeres sparsely fringed beneath; scape long and thin, lacking cicatrix, the 3^rd^ antennomere 1.8–2.0 times as long as scape, subequal to the 4^th^ antennomere. Pronotum wider than long; with fine transverse grooves at the anterior and posterior margin and transverse premedian and postmedian depressions; lateral spine short to long, acute at apex; prosternal process widened and emarginate at apex, procoxal cavities slightly open posteriorly; mesosternal process without tubercle and obliquely sloped in lateral view; mesocoxal cavities open externally to epimera. Elytra elongate, more than 3 times as long as the head and pronotum united, subparallel-sided, rounded at apex, distinctly wider than the base of pronotum. Leg long and slender, mesotibia with an external oblique groove near apex, tarsus five segmented, tarsal claws divaricate.

#### Diagnosis.

The following combination of characters apparently separate *Microcriodes* from other genera in Batocerini: Antennae smooth, without spinous rugosity or traces of spines on the surface. Antennal scape lacking a distinct cicatrix. Eye with lower lobe longer than broad.

#### Notes.

Breuning (1943) compared this genus with *Abatocera* Thomson, 1878, suggesting that it belongs to the tribe Batocerini. All authors treated *Microcriodes* as a member of the tribe Batocerini (Gilmour and Dibb 1948; Gilmour 1963; Rigout 1982; Nýlander 2004). However, the endophallus is quite different from that of *Apriona* spp. and *Batocera* spp. according to our observations. The tribal treatment based on analysis of endophallus morphology will be discussed in the future.

### Microcriodes sikkimensis

Breuning, 1943

http://species-id.net/wiki/Microcriodes_sikkimensis

Figures 1–4 , 7–10

Microcriodes sikkimensis Breuning, 1943: 15. Type locality: Sikkim. Type depository: NHML.
Microcriodes sikkimensis ; Gilmour and Dibb 1948: 100; Gilmour 1963: 483, pl. 2, figs 4–5; Rigout 1982: 10, pl. 14; Weigel 2012: 408, pl. 28, fig. h.

#### Type material examined.

Holotype, female (sex not mentioned in its original description and misidentified as male by Gilmour 1963), “Sikkim” [white label printed]; “Microcriodes / sikkimensis / mihi Type” (handwritten) / “det. Breuning” (printed) [white label]; “Type” [white label with red circle printed] examined through four pictures taken by Yi-Kai Zhang in NHML.

#### Additional materials examined.

(21 specimens, 14 males and 7 females): China: Xizang (Tibet) Autonomous Region: 4 males, 2 females, Xizang, Motuo County (=Mêdog County), Hanmi, 2100m, 2011.VII.23-31, leg. Wen-Xuan Bi (CBWX); 1 male, same date but 2011.VII.29 (CBWX); 1 female, same date but 2011.VIII.2 (CBWX); 1 female, same date but 2011.VII.23–26, leg. Ye Liu (IZAS); 1 male, same date but 2011.VII.23, leg. Ye Liu (IZAS); 1 male, same date but 2013.VII.18 (CBWX); 1 male, same date but 2013.VII.22 (CBWX); 2 males, same date but 2013.VII.29 (CBWX); 2 males, same date but 2013.VII.30 (CBWX); 1 female, same date but 2,128 m, 2013.VII.13, leg. Xiao-Dong Yang (CCCC); 1 male, same date but 1,989 m, 2013.VII.26, leg. Xiao-Dong Yang (CCCC); 1 female, same date but 2,128 m, 2013.VII.30, leg. Xiao-Dong Yang (CCCC); 1 male, Xizang, Motuo County, 80 K, 2,100 m, 2012.VII.23, leg. Xiao-Dong Yang (CCCC); 1 female, Xiang, Motuo County, 62 K, 2,780 m, 2013.VIII.13, leg. Chao Wu (CBWX).

#### Supplementary description.

**Figures 1–6.**
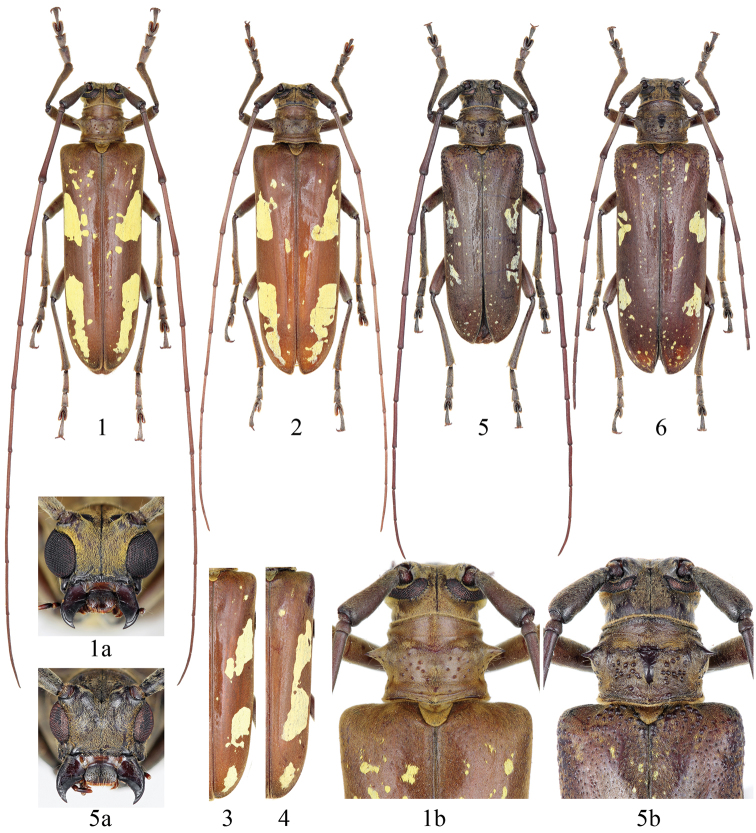
Habitus of *Microcriodes* spp. **1–4** *Microcriodes sikkimensis* Breuning, 1943 **1** male (28.0 mm), from Hanmi, Motuo, Xizang, China **2** female (31.0 mm), from 62K, Motuo, Xizang, China **3–4** right elytron, showing the varieties of maculae **5–6** *Microcriodes wuchaoi* sp. n. **5** holotype (25.5 mm), male, from 62 K, Motuo, Xizang, China **6** paratype (29.5 mm), female, from same locality. **a** head in frontal view **b** pronotum and basal part of elytra in dorsal view, showing granules on pronotal disk and elytral base. Not to scale.

Male (Fig. 1): Length: 21.0–28.0 mm, humeral width: 6.0–9.0 mm. Female (Fig. 2): Length: 30.0–34.0 mm, humeral width: 9.0–10.5 mm. The elytral maculae of this species is variable but generally as follows: besides some scattered small, round, yellow spots, each elytron provided with three main, bright yellow, well-defined, longitudinal but somewhat irregularly shaped maculae on basal one third, a little behind middle and near apex (Fig. 3); the middle macula commonly fused with the hind one as the holotype, but sometimes attach to the front one (Fig. 4). Antenna with 3^rd^ antennomere twice as long as scape, slightly shorter than 4^th^ in male while slightly longer than 4^th^ in female; relative length of antennomeres as follows: male: 5.5 : 1.0 : 11.8 : 12.1 : 11.4 : 10.3 : 10.1 : 9.1 : 8.7 : 8.4 : 11.9; female: 4.7 : 1.0 : 9.9 : 9.4 : 8.0 : 7.1 : 6.4 : 5.7 : 5.6 : 5.3 : 7.1. Pronotum broader than long, 0.8 times as long as basal width, the width across lateral spines about 1.3 times of basal width; lateral spine long, thickened at base with acute apex; disk smooth, with several fine setigerous granules behind middle. Elytra ca. 1.8 times as wide as pronotal base, 2.7 times as long as humeral width, with some very fine granules at base.

**Figures 7–14.**
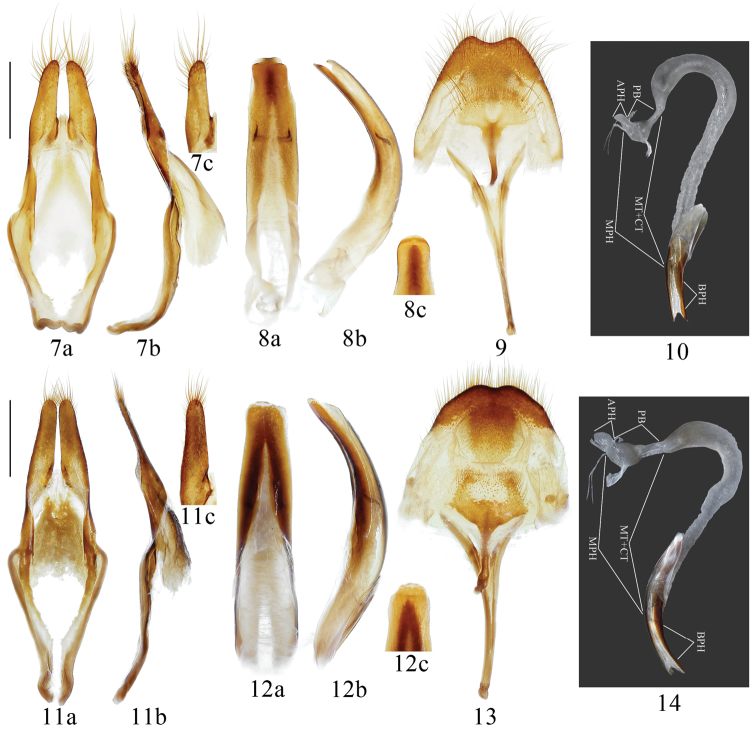
Male genitalia of the *Microcriodes* spp. **7–10** *Microcriodes sikkimensis* Breuning, 1943 **11–14** *Microcriodes wuchaoi* sp. n. **7, 11** tegmen **8, 12** median lobe **9, 13** Tergite VIII with sternites VIII & IX **10, 14** endophallus in non-everted condition. **a** vetral view **b** lateral view **c** antero-dorsal view. Scale 1 mm. **10, 14** not to scale.

**Male genitalia** (Figs 7–10). Tegmen (Fig. 7) in lateral view strongly curved near base, ca. 3.5 mm in length, rhombic in shape and widest behind middle in ventral view; lateral lobes ca. two-ninths of total length of tegmen, provided with long setae on apical half. Median lobe (Fig. 8) slightly shorter than tegmen; moderately curved in lateral view; apex rounded subacuminate in antero-dorsal view. Tergite VIII (Fig. 9) nearly as broad as long, apex distinctly emarginate, with moderately long setae. Endophallus in non-everted condition (Fig. 10) long, about 3 times as long as median lobe, with 3 membranous parts, BPH, MPH and APH; BMP short, about one quarter of the length of median lobe; MPH long, about 2.7 times as long as median lobe, strongly curved at basal two-thirds, with MT and CT fused each other, of which delimited from PB by a distinct constriction; MT+CT sparsely provided with small spicules which become denser near swollen apex; PB provided with same kind of spicules as MT+CT, which become denser at apical half, basal part of PB rather narrow, only 0.35 times as wide as anterior part; APH short, cylindrical in shape. Ejaculatory ducts double.

#### Distribution.

**China** (**new country record**): Xizang (Tibet) Autonomous Region; **India:** “Sikkim” (Breuning 1943), Arunachal Pradesh (Weigel 2012).

### Microcriodes wuchaoi sp. n.

http://zoobank.org/E15E3E47-7555-490D-AF0E-B937ABB0E6E2

http://species-id.net/wiki/Microcriodes_wuchaoi

Figures 5–6 , 11–14

#### Type material.

**Holotype:** male, “China: Xizang, Motuo, 62K / 2013.VIII.9 / 2780 m / leg. Chao Wu” [white label printed] (SNUC). **Paratype:** 1 female, “China: Xizang, Motuo, 62K / 2013.VIII.10 / 2780 m / leg. Chao Wu” [white label printed] (SNUC).

#### Description.

**Male** (Fig. 5). Body length 25.5 mm, humeral width 8.0 mm. Body dark brown; most of ventral surface and legs evenly covered with dense grayish yellow pubescence. Head with mandible (base and outer face), frons, gena and vertex densely covered with grayish yellow appressed pubescence. Vertex with two vittae behind upper eyelobes only sparsely pubescent. Antenna with scape covered with same kind of pubescence as head; other parts covered with fine grayish pubescence. Pronotum covered with same kind of pubescence as head except for a median longitudinal glabrous area. Scutellum densely clothed with recumbent pubescence. Elytron densely covered with grayish yellow appressed pubescence, provided with two bright yellow, irregularly shaped maculae on basal one-third and basal two-third near lateral margin; with small, round, yellow spots scattered mainly around suture and near apex.

Body elongate, subcylindrical, feebly narrowed posteriorly. Head nearly as wide as pronotal width at base, occiput with several granules anteriorly; eyes emarginate, coarsely faceted; lower eye lobe twice as long as width, about twice as long as gena (Fig. 5a). Antenna long and thick, about 1.8 times as long as body length, approximately surpassing elytral apex at tip of sixth antennomere; scape gradually thickened apically; scape and basal third of 3rd antennomere sparsely fringed beneath by suberect short setae; 3rd antennomere 1.8 times as long as scape, subequal to 4^th^ and 5^th^; relative length of antennomeres as follows: 4.9 : 1.0 : 8.7 : 8.7 : 8.5 : 7.5 : 7.4 : 6.4 : 5.8 : 4.6 : 5.5.

Pronotum broader than long, 0.8 times as long as basal width, the width across lateral spines about 1.25 times of basal width; lateral spine short, thickened at base with acute apex; disk with a posteromedial longitudinally oval callus, provided with several distinct setigerous granules at both sides of callus and behind lateral spines.

Scutellum obtuse-triquetrous. Prosternum with prosternal process widened apically; procoxal cavities closed posteriorly. Mesosternal process without tubercle and obliquely sloped in lateral view.

Elytra ca. 1.8 times as wide as pronotal base, 2.4 times as long as humeral width, very slightly convergent toward apices; basal tenth of elytron provided with distinct round granules near suture and humerus, respectively; disk moderately punctured, becoming more shallow near apices.

Leg moderately slender, mesotibia with an external oblique groove near apical third, metatibia reaching elytral apex at apical one-fourth.

**Male genitalia** (Figs 11–14). Tegmen (Fig. 11) in lateral view moderately curved, ca. 3.8 mm in length, rhombic in shape and widest near middle in ventral view; lateral lobes ca. one-fourth of total length of tegmen, which moderately provided with short setae on apex. Median lobe (Fig. 12) shorter than tegmen; gently curved in lateral view; apex emarginate in antero-dorsal view. Tergite VIII (Fig. 13) slightly broader than long, apex slightly emarginate with short setae. Endophallus in non-everted condition (Fig. 14) moderately long, about 2.7 times as long as median lobe, with 3 membranous parts, BPH, MPH and APH; BMP short, about one-third length of median lobe; MPH long, about 2.1 times as long as median lobe, strongly curved at basal two-fifths, with MT and CT fused with each other, of which delimited from PB by a moderate constriction; MT+CT slightly swollen at apical tenth, sparsely provided with small spicules which are getting denser at apical one-fifth; PB provided with same kind of spicules as MT+CT, which are getting denser at apical half; basal part of PB moderately narrow, ca. 0.5 times as wide as anterior part; APH short, moderately swollen at middle and rounded at apex. Ejaculatory ducts double.

**Female** (Fig. 6). Body length 29.5 mm, humeral width 9.4 mm. Almost identical to male in general appearance. Antenna about 1.1 times as long as body, surpassing elytral apex at the middle of tenth antennomere; relative length of antennomeres as follows: 4.6 : 1.0 : 7.4 : 6.5 : 5.7 : 4.9 : 5.0 : 4.4 : 3.7 : 3.2 : 3.8. Leg slightly shorter, metatibia hardly reach elytral apex.

#### Diagnosis.

This new species resembles *Microcriodes sikkimensis* Breuning, 1943 by the general habitus, but is distinguishable from the latter by combination of the following characters: color of integument darker; elytron shorter in relation to the body length; antenna shorter and thicker; lower eye lobe narrower; pronotal lateral spine shorter; pronotal disk with a shiny posteromedial callus; distinctly granulated on pronotal disk and elytral base; elytron with the main maculae relatively short and small, lacking a bright yellow macula near apex.

#### Etymology.

The new species is dedicated to its discoverer, Mr. Chao Wu. We use the Chinese format “Wu +Chao” (family name + first name) for this name.

#### Distribution.

China: Xizang (Tibet) Autonomous Region.

## Supplementary Material

XML Treatment for Microcriodes

XML Treatment for Microcriodes sikkimensis

XML Treatment for Microcriodes wuchaoi
